# Effect of COVID-19 Pandemic-Induced Dietary and Lifestyle Changes and Their Associations with Perceived Health Status and Self-Reported Body Weight Changes in India: A Cross-Sectional Survey

**DOI:** 10.3390/nu13113682

**Published:** 2021-10-20

**Authors:** Jagmeet Madan, Traci Blonquist, Eram Rao, Ankita Marwaha, Joshya Mehra, Richa Bharti, Nishi Sharma, Ritika Samaddar, Sandhya Pandey, Eunice Mah, Varsha Shete, YiFang Chu, Oliver Chen

**Affiliations:** 1 Sir Vithaldas Thackersey College of Home Science (Autonomous), Shreemati Nathibai Damodar Thackersey Women’s University, Mumbai 400049, India; dr.jagmeetmadan@gmail.com; 2 Biofortis Research, Merieux NutriSciences, Addison, IL 60101, USA; traci.blonquist@mxns.com (T.B.); eunice.mah@mxns.com (E.M.); 3 Department of Food Technology, Bhaskaracharya College of Applied Sciences, University of Delhi, New Delhi 110075, India; eramrao@gmail.com; 4 PepsiCo, Inc., Gurugram 122022, India; Joshya.mehra@pepsico.com (J.M.); Richa.Bharti@pepsico.com (R.B.); Nishi.sharma@pepsico.com (N.S.); 5 Max Healthcare, New Delhi 110017, India; rsamaddar@maxhealthcare.com; 6 Medanta, New Delhi 110024, India; Sandhya.Pandey@Medanta.org; 7 PepsiCo, Inc., Barrington, IL 60010, USA; Varsha.Shete@pepsico.com (V.S.); yifang.chu@pepsico.com (Y.C.); 8 Friedman School of Nutrition Science and Policy, Tufts University, Boston, MA 02111, USA

**Keywords:** age, diet, gender, physical activity, sleep

## Abstract

Home confinement during the COVID-19 pandemic is accompanied by dramatic changes in lifestyle and dietary behaviors that can significantly influence health. We conducted an online cross-sectional survey to assess COVID-19 pandemic-induced dietary and lifestyle changes and their association with perceived health status and self-reported body weight changes among 1000 Indian adults in early 2021. Positive improvements in dietary habits, e.g., eating more nutritious (85% of participants) and home-cooked food (89%) and an increase in overall nutrition intake (79%), were observed. Sixty-five percent of participants self-reported increased oat consumption to support immunity. There were some negative changes, e.g., more binge eating (69%), eating more in between meals (67%), and increasing meal portion size (72%). Two-thirds of participants reported no change in lifestyles, whereas 21 and 23% reported an increase, and 13 and 10% reported a decrease in physical activity and sleep, respectively. Overall, 64 and 65% of participants reported an improvement in perceived health and an increase in body weight during the COVID-19 period compared to pre-COVID-19, respectively. The top motivations for improving dietary habits included improving physical and mental health and building immunity. In conclusion, the overall perceived health was improved and there was an increase in self-reported body weight in most participants during COVID-19. Diet emerged as the most crucial determinant for these changes.

## 1. Introduction

The implementation of measures, such as closing borders, home confinement, quarantine, and physical (social) distancing, are crucial for controlling pandemics such as COVID-19, which has posed a global public health emergency threat since early 2019. Despite their apparent benefits to safety and health related to pandemics, these measures can have a significant repercussion on health and well-being because they can drastically and suddenly alter lifestyle behaviors of people in confinement, such as diet, physical activity, smoking, mealtime, sleep pattern, and alcohol use [1,2,3,4,5].

During the COVID-19 pandemic, particularly when a home confinement policy was implemented by governments, people may have harder access to healthy foods and fitness facilities. They may also start habits that are associated with negative health impacts, such as more time spent watching TV and increased energy intake [6,7]. A study conducted on 37,252 French adults showed that food intake increased, but diet diversity, intake of fresh produce, and physical activity decreased during the COVID-19 confinement between April and May 2020 [8]. Phillipou et al. [9] also noted an increase in both food consumption restrictions and binge eating behaviors in the general population in Australia. Additionally, long-term isolation from staying home can negatively impact mental health, potentially leading to disorders such as depression and anxiety. The consequences of these behavior changes can be impactful if the negative habits established during this challenging time remain after lifting of the COVID-19-related policies.

On the other hand, during the current COVID-19 pandemic, positive lifestyle behavior changes have emerged in some populations because of increased time at home and the threat of becoming ill with COVID-19 [10]. For example, a proportion of Italian respondents to an online survey conducted in April 2020 increased resistance exercise, had a greater adherence to the Mediterranean diet, and began consuming organic produce [2]. Similarly, increased adherence to the Mediterranean diet was observed in some people in Spain [11].

Diet and lifestyle behaviors are critical factors in health promotion and prevention. Eating a healthy diet and frequently participating in the recommended amount of physical activity are associated with reduced all-cause mortality and improved health and well-being [12,13]. Lack of physical activity and changed mealtime and portion size can all contribute to an increased likelihood of body weight (BW) gain and health deterioration [8,14]. The development of negative or positive habits during COVID-19 confinements may be due to various individual (e.g., motivation, intention, mental state), sociodemographic (e.g., age, gender, employment status, family responsibilities), and environmental (e.g., access to healthy foods, fitness facilities) factors [15,16]. Many of these potential factors are disrupted by the COVID-19 pandemic; thus, the current pandemic provides a natural experimental setting to assess not only how a global pandemic affects diet and lifestyle behaviors but also how changes in these behaviors affect health outcomes such as BW gain or loss as well as self-perceived health, which is an important factor influencing the decision to engage in positive health behaviors [16,17]. Understanding these underlying factors is important in refining current strategies that promote health-protective behaviors.

Various studies [1,2,4,5,8,9,10,11,14,18,19] have assessed the effect of the COVID-19 pandemic on diet and lifestyle, but pandemic restrictions that are implemented to control the transmission of COVID-19 vary between countries, depending on the severity of the virus transmission. The impact of the restrictions on lifestyle behaviors and changes in health is anticipated to be different between populations, and thus, population-specific investigations are warranted.

The primary objective of this online survey study was to assess whether there were changes in dietary and lifestyle behaviors and key health concerns, as well as investigate possible associations with perceived health status and BW changes during the COVID-19 pandemic in India. We hypothesized that the COVID-19 confinement in India would result in lower diet quality and promote negative lifestyle behaviors, which would be associated with increased BW gain and poor perceived health.

## 2. Materials and Methods

### 2.1. Survey

A prospective cross-sectional online survey among adults in the A and B Socio-Economic Classification (SEC) segments, aged 18–50 years (inclusive), who resided in the four largest metro cities (Delhi, Mumbai, Bangalore, and Kolkata) in India, was conducted to describe changes in perceived health, self-reported BW and dietary and lifestyle behaviors during the COVID-19 period to their pre-COVID-19 routines. Other participant criteria included work status (students, salaried, self-employed, and homemaker (minimum 200 each)) and household structure (staying alone/with friends, nuclear families without kids, nuclear families with kids, and joint family (minimum 150 each)). The study was performed in compliance with ICH E6R2 “Guidance on Good Clinical Practice”, Indian Good Clinical Practices Guideline, ICMR Ethical Guidelines for Biomedical Research on Human Subjects, ICMR National ethical guidelines for biomedical and health research involving human participants 2017, and in accordance with the Declaration of Helsinki. All participants were free to withdraw their participation from the study at any time, for any reason, specified or unspecified, and without prejudice to further treatment. Participants were fully informed about the objective of the study and were asked for permission to use and publish the data. Before starting the online survey, all participants had to provide electronic informed consent to participate in the study. Participants were compensated for completing the survey. The study protocol, including the informed consent form, was approved by Institutional Ethics Committee Jehangir Clinical Development Center Pvt. Ltd. (Prune, India). Euromonitor International administered this survey through Toluna Inc. (Gurgaon, India), a leading panel provider, who abided by the ICC/ESOMAR code for ethical market research.

A sample size of 1000 participants was selected based on a 95% confidence level (CI) with a width of ±3.1%, which was considered statistically significant for research purposes under non-random quota sampling. Participants were divided equally among the four largest metro cities (Delhi, Mumbai, Bangalore, and Kolkata) in terms of population and GDP contribution. Total urban population in India was 457,419,800 (35% of total population) in 2019 [20]. The median age of the urban population was 28.3 years, and 55% of the population fell between 18 and 50 years (6). Thus, the participants were also sampled by gender (male vs. female = 1:1) and age (18–25 (Generation Z) vs. 26–40 (Millennials) vs. 41–50 (Generation X) = 4.5:4.5:1), using a non-random quota sampling via an online panel to ensure an even and representative distribution.

The data collected were categorical in nature, which were presented with counts and percentages for both pre- and during-COVID-19 periods. A web-form survey (Survey Questions S1) consisting of 34 questions was used to collect lifestyle habits, eating behaviors, self-reported BW, and health and well-being conditions. For example, the survey collected the amount of time participants spent on 11 lifestyle activities (e.g., doing professional work, participating in physical exercise, engaging in leisure entertainment activities, sleeping, traveling in the city), the timing of meals and snacks, skipped meals, frequency and servings of 14 food groups (e.g., fruits, vegetables—salad, eggs, cooked roots/tubers, cooked green leafy vegetables, chicken/mutton/fish/prawn, milk/curd/paneer, whole wheat, cooked white rice), rating of health conditions, and perceived BW change. These 14 food groups were selected for the assessment because they are typically consumed in India. Prior to survey distribution, the content validity of the questionnaire was assessed based on the review of six subject experts not related to the conduct of this survey. Specifically, ten questions in the main questionnaire, which were identified on the basis of direct correlation with the objectives of the study, were rated by each expert for its relevance (1 = very irrelevant, 2 = irrelevant, 3 = relevant, and 4 = very relevant). The content validity index for each question was calculated by dividing the number of raters who scored the item as 3 or 4 by the total number of raters. All ten selected questions scored above 0.8 and thus, were considered adequate [21]. The total completion time for the survey was estimated at 15 min. The survey was launched on 21 January 2021 and participants completed the survey by 10 February 2021. All the surveys were anonymously collected and processed for statistical analyses.

### 2.2. Statistical Analysis

All survey response data were summarized as counts and percentages. Lifestyle and dietary intake items that were queried for the pre-COVID-19 and during-COVID-19 period were collapsed into three categories and compared using Bowker’s symmetry test, and a false discovery rate (FDR) adjustment was used for each set of lifestyle and dietary intake items. Response categories for the frequency of consumption of the specified food groups were grouped to (1) less than 3 times a week, (2) 4–5 times a week to twice a day, and (3) 3 or more times a day. The lifestyle parameter for the time being physically active was grouped to (1) less than 30 min, (2) from 30 min to 3 h, and (3) more than 3 h. Time sleeping was grouped to (1) less than 5 h, (2) 6–8 h, and (3) more than 8 h. All other lifestyle parameters were grouped to (1) less than 1 h, (2) 1–5 h, and (3) 6 or more hours. The association of changes in dietary intake with BW change perception, health change perception, gender, generation (categorized by collapsing age into three age groups defined as generation), and location was explored with a chi-square test. An FDR adjustment was used.

The factors related to the participant’s perceived improvement in health and increase in BW, compared to the pre-COVID-19 months, were explored with classification trees due to the number of candidate factors and the possible relationship between factors. Classification trees involve a non-parametric approach to explore complex relationships between candidate factors and an outcome of interest. Considered factors included generation (age group), gender, city, changes in dietary and lifestyle habits, and concern for health conditions during the COVID-19 period. Recursive partitioning was used to build the classification trees, exploring the most influential factors related to the following outcomes: (1) an improvement in health versus deterioration/no change and (2) an increase in BW versus a decrease/no change, using the rpart function in R [22,23]. The first split indicates the factor that best classifies the data into the appropriate outcome groups. Further splits may occur until no other factors adequately contribute to the classification of the outcome of interest. Trees were pruned such that the number of splits with the minimum cross-validated error was retained.

All analyses were performed in SAS Version 9.4 and R version 4.0.3.

## 3. Results

### 3.1. Respondent Characteristics

Among the 1000 participants, 50% were male, 45% were aged between 18 and 25 years or between 26 and 40 years old, and 78% achieved a graduate/post-graduate education level (Table 1). Participants were recruited evenly (25% from each location) from four cities representing each region of India: Bangalore, Dehli/NCR, Kolkata, and Mumbai. A minority (~2%) of the survey sample lost employment during COVID-19, and less than 2% reported living alone.

### 3.2. Health and Nutrition Status—Perception and Concerns

Overall, 79% of the participant’s self-rated health was good to excellent. Sixty-four percent (95% CI 60–67%) indicated a perceived improvement in health during the COVID-19 period compared to pre-COVID-19, and 14% (95% CI 12–16%) indicated a perceived deterioration. A total of 65% (95% CI 62–68%) indicated an increase, and 15% (95% CI 12–17%) indicated a decrease in self-reported BW during the COVID-19. In identifying the factors related to the perceived improvement of health during the COVID-19 period, the most important factor identified with the pruned classification tree was the perceived increase in nutrition intake overall, with a classification rate of 0.70. The most important factor identified with the pruned classification tree in identifying the factors related to perceived BW gain during the COVID-19 period was the perceived increase in portion size with a classification rate of 0.74.

A majority (>60%) of participants were concerned for each of the queried health conditions (stress/anxiety, obesity/weight management, depression/mental health, physical tiredness and fatigue/low energy, lack of immunity, digestive issues, virtual fatigue, sleeping problems, heart health and heart disease, cholesterol issues, hypertension, joint pain, diabetes, and women’s health), where the five most common included reporting concern about stress/anxiety (72.2%), obesity/weight management (71.2%), depression/mental health (69.8%), fatigue/low energy (69.6%), and lack of immunity (69.6%) (Supplemental Table S1). The proportions reporting a concern about obesity and immunity appeared comparable between genders and generations. Out of 771 participants reporting a concern for BW, the most common diet regimens tried during the COVID-19 period included eating home-cooked food only (48%), low carbohydrate/high protein (44%), intermittent fasting (37%), and keto diet (33%) (Supplemental Table S2).

### 3.3. Dietary Habits

Participants were asked to rate the change of dietary habits during the COVID-19 period, and responses were collapsed to agree, neutral, and disagree. Positive improvements in queried dietary habits include an increase in overall nutrition intake (79%), eating healthier food (85%), eating more home-cooked food (89%), keeping track of what/how much eaten in a day (74%), starting cooking at home more often (84%), and started looking for healthy recipes to cook at home (85%). Negative changes include binge eating more (69%), eating more snacks in between meals (67%), an increase in portion size of a meal (72%), and feeling discouraged to cook (58%). There was a smaller proportion (28%) of participants changing eating habits for BW. A significant association was suggested between gender and self-reported eating between meals (*p* = 0.0018) and feeling discouraged to cook (*p* = 0.0010), where a higher proportion of negative behavior was suggested for males. A significant association was also suggested between gender and self-report of starting to cook at home more often (*p* = 0.0023), where a higher proportion of positive behavior was suggested for males. No other associations between these positive and negative behavior changes were detected for gender or generation when adjusting for multiple comparisons.

**Figure 1:**
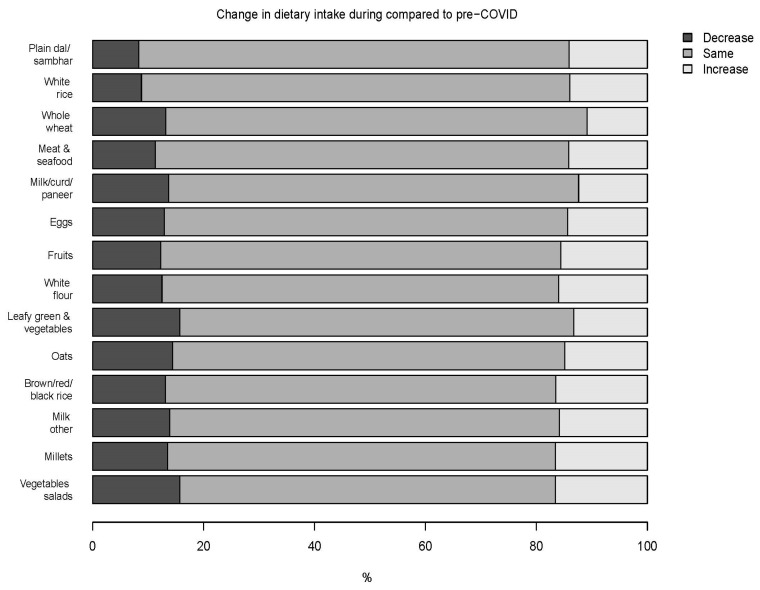
Percentage of participants reporting a decrease, no change (same), or increase in intake frequencies of 14 food groups during the COVID-19 confinement compared to pre-COVID-19.

A high percentage (>68%) of participants tended to maintain the intake frequency of 14 food groups during the COVID-19 period as compared to pre-COVID-19 (Figure 1, Supplemental Table S3). The test of symmetry was significant in the frequency of intake for cooked millets (*p* = 0.046), fruits (*p* = 0.024), meat and seafood (*p* = 0.042), plain dal/sambhar (*p* = 0.002), and white rice (*p* = 0.007) (Table 2). For each of these food groups, there was a trend toward more intake. However, only intake of plain dal/sambhar and white rice remained significant after adjustment for multiple comparisons. No other significant changes were noted.

**Figure 2:**
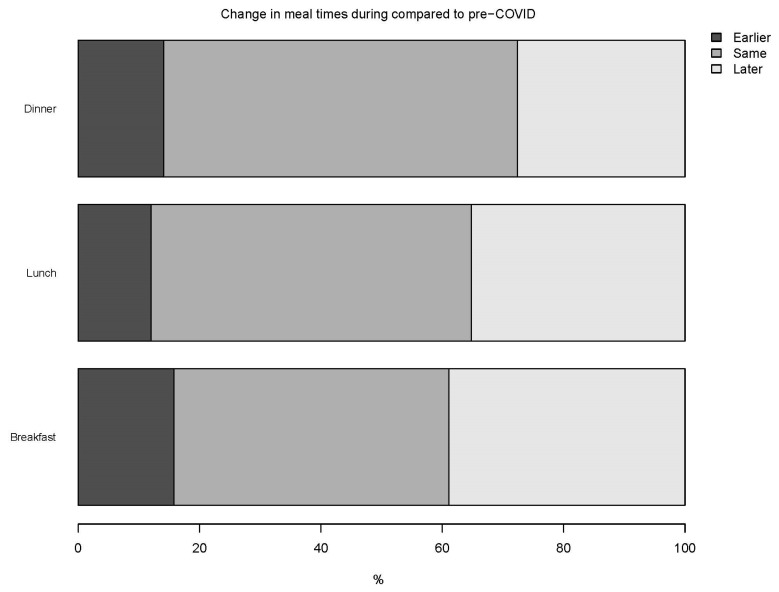
The percentage of respondents’ change in mealtimes for breakfast, lunch, and dinner during the COVID-19 confinement compared to pre-COVID-19.

With respect to mealtimes, nearly 39% were consuming breakfast later during the day, while 35% and 28% were consuming late lunch and dinner, respectively (Figure 2). A high percentage of participants reported no change from pre- to during the COVID-19 period in the time of breakfast (45%), lunch (53%), and dinner (58%). In relation to eating breakfast, 72% of participants indicated it gave energy for at least half the day, and 60% indicated it made them feel full longer. Additionally, 68% of Millennials reported breakfast makes them feel full longer compared to 51% of Generation Z and 61% of Generation X. In general, if a meal was skipped, 44% of participants reported a tendency to skip breakfast more often than other meals. Of those that reported they might skip breakfast, the top three reasons included a late start due to work schedule (51%), not being hungry enough (42%), and not having enough time (35%). The tendency to skip breakfast was associated with age group (*p* < 0.001), where a higher proportion of Generation X (49%) and Millennials (49%) might skip breakfast compared to Generation Z (38%). No association with skipping breakfast was indicated by gender (*p* = 0.14). In total, 197 (18%) of participants reported more snacking periods, and 83 (8%) reported fewer snacking periods during the COVID-19 period than pre-COVID-19. In both the pre- and during-COVID-19 periods, the most common snacking time (~85%) was between lunch and dinner.

### 3.4. Oat Consumption

**Figure 3:**
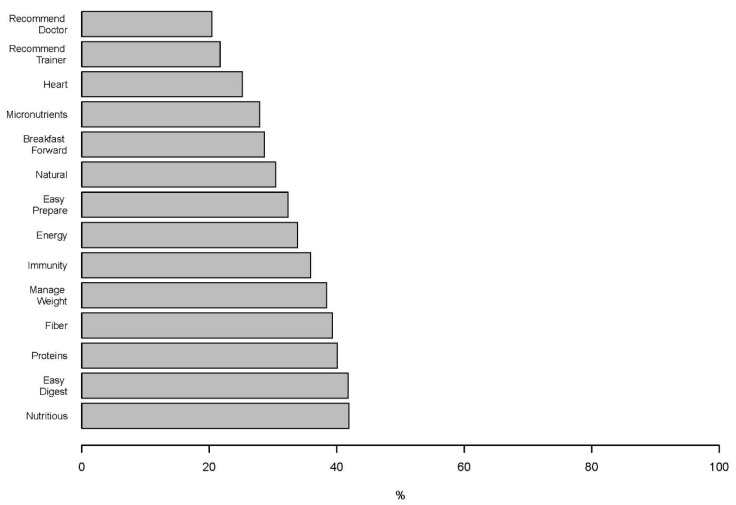
Top factors reported by percentage of participants contributing to oat consumption during the COVID-19 confinement.

Sixty-five percent of participants reported consuming oats, in any form, at least once a day during the pre-COVID-19 period. Only 7.9% of participants reported never consuming oats during the pre-COVID period. Of the participants reporting any consumption of oats prior to the COVID-19 pandemic, 83% indicated eating for breakfast, and 71% reported preferring to consume oats as a main dish. The five most selected reasons for consuming oats included nutritious and healthy (41.9%), easy to digest (41.8%), rich in protein (40.1%), rich in fiber (39.3%), and help manage BW (38.4%) (Figure 3). Additionally, 35.9% indicated oats are consumed to help strengthen immunity. To support immunity during the COVID period, 64.4% (95% CI 61.3–67.4%) self-reported an increase in oat consumption in any form. Of those that responded, there was a significant association between the self-reported change in oat consumption and generation (*p* = 0.034), whereby fewer participants in Generation Z (59.5%) increased intake compared to Generation X (66.0%) and Millennials (68.8%). No significant association was detected with gender. When querying the serving size of cooked oats, 70.7% (95% CI 67.8–73.5%) of participants reported no notable changes from pre- to during-COVID-19 period.

### 3.5. Lifestyle Behavior

A majority (>60%) of participants did not drastically change any of the 11 lifestyle parameters queried. Approximately 55% of participants reported no change, and 32% reported a decrease in the amount of time traveling in the city for work, school, or shopping. For all of the queried lifestyle parameters, except time socializing with family or roommates, the test of symmetry was significant (*p* < 0.001), implying a shift in time spent doing various lifestyle parameters from pre- to during the COVID-19 period. The time for being physically active, sleeping, cleaning, connecting virtually, cooking, doing leisure entertainment activities, being on social media, and watching recipe-related videos tended to increase more often than it decreased from pre- to during the COVID-19 period. Approximately 66 and 67% of participants reported no change in time for physical activity and sleep, respectively, but 21 and 23% of these reported an increase as compared to 13 and 10% who reported a decrease. In comparison, the time spent doing professional work and traveling in the city tended to decrease, rather than increase, from pre- to during the COVID-19 period (Table 3).

**Figure 4:**
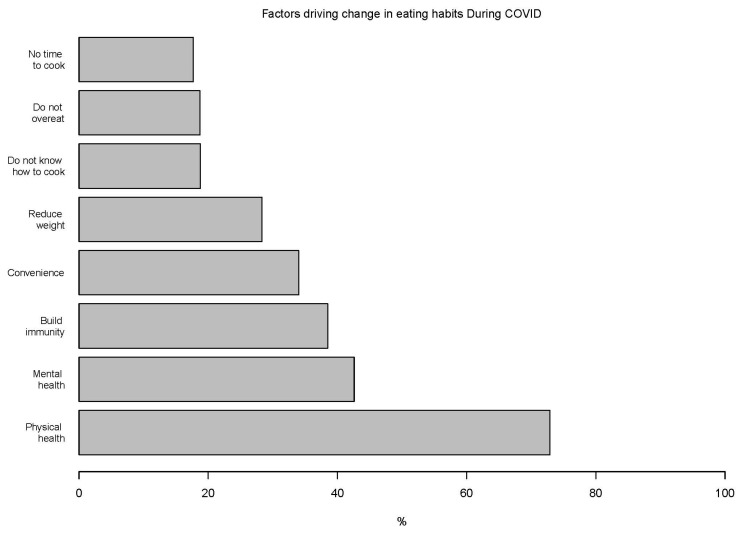
Top factors reported by percentage of participants contributing to a change in dietary habits.

During the COVID-19 period, the most common physical and mental health regimens initiated were yoga (58%), walking/running (56%), breathing exercises (52%), and meditation (43%) (Supplemental Table S4). The top factors contributing to a change in eating habits during the COVID-19 period included improving physical health (73%) and mental health (43%) and building immunity (38%) (Figure 4).

### 3.6. Associations with Perceived Health and Self-Reported BW Change

No statistically significant association was detected between self-reported BW change and gender (*p* = 0.46) or generation (age group) (*p* = 0.08). However, self-reported BW change was associated with the location. A higher proportion of participants in Bangalore reported a decrease in BW, whereas a higher proportion in Delhi/NCR reported an increase in BW. No significant association was detected between perceived health change and gender (*p* = 0.11) or city (*p* = 0.07). Generation (age group) was significantly associated with perceived health change (*p* = 0.014), where Generation Z reported a smaller proportion for the improvement and larger proportion for the deterioration than the other two generations.

**Figure 5:**
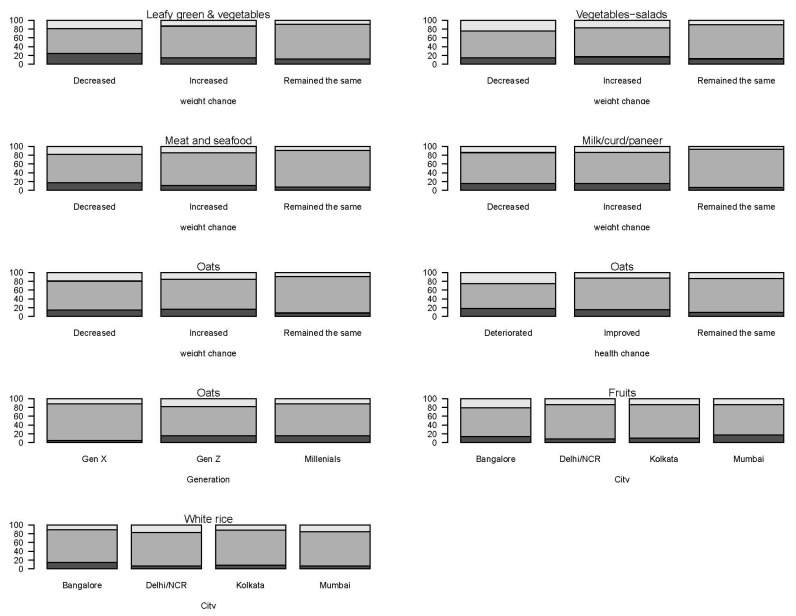
The percentage of participants reporting a decrease (dark gray), no change (medium-gray), or increase (light gray) during the COVID-19 confinement compared to pre-COVID-19 in dietary intake within self-reported body weight change, perceived health change, generation, and city if significant after multiple comparison adjustments (*q* < 0.05).

Associations between dietary changes and perceived health and self-reported BW changes, gender, generation, and city were assessed (Supplemental Table S5). There were significant associations (FDR *q*-value < 0.05) between self-reported BW change and the change in dietary intake for cooked leafy greens and vegetables, vegetable—salads, meat and seafood, milk/curd/paneer, and oats (Figure 5). Of those that reported a decrease in reported BW, more participants changed the amount of cooked leafy greens and vegetables and oats (either increase or decrease) compared to those that increased or did not report BW change. Of those that self-reported BW remaining the same, the intake of raw vegetables-salads, meat and seafood, milk/curd/paneer, and oats also tended to remain the same, whereas consumption fluctuated for those with self-reported BW decreasing or increasing.

Perceived change in health status and generation (age group) were also associated with the change in eating oats only (FDR *q*-value < 0.05) (Figure 5). Participants who reported a deterioration in health more often changed oats intake (either increase or decrease) compared to those who reported an improvement or no change in health. City was significantly associated with the change in intake of fruits and white rice (FDR *q*-value < 0.05) (Figure 5). A higher proportion indicated intake of fruit remained the same in Delhi/NCR and Kolkata compared to Bangalore and Mumbai, and a higher proportion indicated a decrease in the intake of white rice in Bangalore. No significant association between gender and dietary changes was detected.

Associations between lifestyle changes and perceived health and self-reported BW changes, gender, generation, and city were assessed. There was a significant association (FDR *q*-value < 0.05) between perceived health change and the change in travel in the city. Of those that indicated that their health remained the same, 3% increased travel compared to 15% for each of those with perceived deteriorated and improved health. No other significant associations were detected with lifestyle changes.

## 4. Discussion

Stay-at-home orders are an essential approach to mitigating the COVID-19 pandemic but are not without repercussions on people’s daily activities, including work, sleep pattern, mealtime, education, social interactions, exercise, and other essential daily tasks, such as buying primary necessities [4,5,24,25,26]. All these changes can, in turn, affect the physical and mental health and well-being of some people. We examined self-reported changes in dietary habits, lifestyle behaviors, perceived health, and BW during the COVID-19 period in India. Compared to pre-COVID-19, the majority of the participants reported improved perceived health, but almost the same proportion reported self-reported BW gain during the COVID-19 period. These were associated with changes in dietary habits such as enhancing overall nutrition intake, eating healthier food, and increasing binge eating and portion size of meals.

Perceived or self-rated health is commonly used in population surveys to assess general health status or health-related quality of life [17]. Changes in lifestyle and dietary habits resulting from stay-at-home orders during the COVID-19 period can affect perceived health status since this can be influenced by psychological well-being and access to adequate nutrition and exercise space or equipment [27,28,29]. A large survey conducted in China in the lockdown of the early COVID-19 outbreak showed that almost 35% of the participants experienced psychological distress [30,31]. In another survey study in Spain, three out of ten participants reported worsening of their physical and mental health during COVID-19 [18]. The negative impact of COVID-19 lockdown on subjective well-being and health was also reported in Germany [32]. In contrast, our results from the survey in India suggest that perceived health status did not deteriorate during the COVID-19 pandemic compared to the status before the pandemic but improved in most of the participants. However, the majority of participants had a concern about mental health, BW management, low energy, lack of immunity, and digestive issues. This observation appears in line with the results of a cross-national online survey administered during the early phase of the COVID-19 pandemic in April 2020, which showed that 41% of participants in India rated their health status high as compared to much smaller percentages in China, Italy, and Japan [19]. The potential differences in perceived health between countries reported in the literature may be ascribed to cultural disparities, self-perceptions, demographic variances, length of confinement, virtual access to interpersonal connections, employment status, and severity of the COVID-19 pandemic.

Many determinants are associated with perceived health, including nutrition, health condition, race/ethnicity, income, education, food security, smoking, exercise, poor mental and physical health, prescription drug use, and presence of chronic disease [33]. A cross-national longitudinal study using machine learning predicted that perceived health during the COVID-19 pandemic was associated with perceived vulnerability to disease (e.g., COVID-19 infection), physical exercise, attachment anxiety, and age [34]. Our study showed that overall nutrition and oat intakes were associated with perceived health status. The main motivation for increasing oat consumption was to support immunity since lack of immunity was one of the main health concerns among the study participants. Even though the age structure of a population might contribute to differences in health perception [35,36], improved perceived health in our study might not be ascribed to a younger age because 57% of participants reporting a decline in perceived health (*n* = 142) were in Generation Z. The age influence on the perceived impact of COVID-19 on psychological outcomes was also not observed in Americans [37]. It is worthwhile to examine underlying factors for the association and health implications. Moreover, future studies are warranted to determine whether good perceived health status in India is linked with lower COVID-19 related mortality than other countries, such as the United States [38].

The COVID-19 pandemic can be linked with BW gain due to disruption in lifestyle and dietary habits, impaired mental health, and increased isolation time. A growing body of survey evidence showed self-reported BW gain during the COVID-19 pandemic compared to pre-COVID-19 [39]. Additionally, a worldwide 67-country online survey with 10,121 participants showed a small but significant increase in body mass index (BMI) from 24.71 to 24.78 kg/m² during the COVID-19 pandemic [40]. Similarly, 5–10 pounds of BW gain was reported by <30% of adult participants during the self-quarantine in the United States [41,42]. Self-reported BW gain during COVID-19 pandemic was also reported in Spain (37.3%) [43], Italy (48.6%) [2], Chile (38.1%) [44], and France (35%) [8]. In our study, the majority of the participants reported increased self-reported BW gain during the COVID-19 period compared to pre-COVID-19. This BW gain can be of concern because a small but significant increase in a relatively short period can lead to a large undesirable BW gain over decades [45].

In a scoping review, Chew and Lopez [39] summarized that the predictors of self-reported BW gain during COVID-19 confinement included being middle-aged, female, higher baseline BMI, increased total food consumption, consumption of junk food, eating in response to the sight and smell of food, stress eating and eating after dinner, sedentary behavior, low water consumption, and fewer sleep hours. Consistently, we found self-reported BW gain was linked with increased food portion size. In contrast, BW gain was not associated with gender, even though it was reported that women were at increased risk for BW gain and sedentary lifestyle during the COVID-19 pandemic due to the more considerable societal strain and burden endured by women [46]. Similarly, a survey of adults in Poland did not show a link between gender and BW change [47]. Our study showed that a small proportion of participants reported a self-reported BW loss, even though the majority of the participants reported a concern about BW. This result is consistent with an Italian survey study finding, in which 19% reported BW loss during the COVID-19 pandemic [48]. Our participants who reported a concern of BW tried regimens such as eating homemade cooked food only, low carbohydrate/high protein, and keto diet or practicing intermittent fasting. Additionally, those who reported increased or decreased BW fluctuated in their intake of vegetables—salads, meat and seafood, milk/curd/paneer, and oats, while those who reported unchanged BW tended not to alter their food group intakes. This observation appears to suggest that a healthy balanced diet that is sustainable for individuals may be helpful for BW control.

Healthy nutrition can be affected by social distancing and stay-at-home order during the COVID-19 pandemic [49]. Additionally, this challenging time can foster unfavorable dietary behaviors due to mental distress or stay-at-home boredom, such as binge eating and drinking and more meals [49,50]. All these factors can link to increased overall energy intake and consequent BW gain. Interestingly, in an international survey in Ibero-American countries, including Brazil, Argentina, Peru, Mexico, and Spain, most participants (62%) did not alter their dietary habits, and 23% tended to eat a healthy diet with a smaller meal size during the COVID-19 pandemic [51]. In contrast, Rodriguez-Perez et al. [11] noted that the adherence to the Mediterranean diet was increased in Spain, suggesting a positive impact on health. Similarly, we noted that the participants in our survey reported perceived improvement in several dietary habits, such as improved overall nutrition intake, eating healthier foods and home-cooked foods, and tracking food consumption. Notably, eating healthier foods was confirmed by the increased proportion of participants reporting eating millets, fruits, and plain dal/sambhar more frequently. Additionally, 64% of the study participants reported an increase in oat consumption during the COVID-19 period. These positive changes may be partly attributed to the intention to improve physical and mental health and build immunity, as reported by the participants in the present survey. However, mixed changes in dietary habits were also observed between populations. In a scoping review, Bennet et al. [10] summarized that 11 studies showed an increase in fresh produce intake and home cooking and a reduction in comfort foods. On the other hand, almost an equal number of studies reported the opposite. Similarly, a small study with 50 participants in Mumbai, India, showed that intakes of fruits and vegetables decreased in more than half participants [52]. Future research should be conducted to examine why some people improved their dietary habits during the COVID-19 pandemic and others did not. Additionally, future studies are warranted to examine what dietary habits people have post-COVID-19.

Meal frequency and timing are linked with disease risk. A regular meal pattern including a larger breakfast and 2–3 meals per day might provide physiological benefits [53]. In contrast, greater meal frequency and late-night eating were associated with metabolic risk factors, such as obesity, hypertension, and hypertriglyceridemia [54,55]. During the COVID-19 pandemic, frequent eating is one way of coping with anxiety and stay-at-home boredom [49]. In a scoping review of 23 studies, Bennett et al. [10] found that six studies reported an increase in meal number and frequency and ten studies reported increased snacking during COVID-19 confinement. In contrast, our study noted that a small proportion of participants reported an increased snacking frequency, but the majority reported no change. However, approximately one-third of participants reported consuming the three main meals at later times during the COVID-19 period as compared to pre-COVID-19. Such a shift in mealtimes was noted in another study with food science students and professionals in Spain [56]. If the new mealtime and frequency are sustained for an extended period, this new diet routine can have marked health concerns, such as cardiometabolic diseases.

Various healthy lifestyle habits, including physical activity, are associated with health span and living without chronic diseases, such as cardiometabolic diseases and cancers [57,58]. During the COVID-19 pandemic, social isolation resulting from stay-at-home confinement appears to change the lifestyles of many people by limiting participation in regular, routine physical activity [59]. The World Health Organization (WHO) has indicated that home confinement should not impede people from participating in physical activities as certain types of exercise can be performed at home [60]. Indeed, a cross-national survey, including China, Japan, India, and Spain, showed that 67.6% of participants (67.8% in India) reported an increased engagement in exercise [19]. Celorio-Sardà et al. [56] showed that in a survey with food science students and professionals in Spain, 49% of participants reported increased frequency of physical activity for at least 30 min but noted that about one-third of the participants reported worsening lifestyle behaviors, including increased sitting time in 67% of participants. However, a decline in physical activity and an increase in sedentary time due to increased time for watching TV or engaging in social media have been observed in the United States [41,61], France [8], Spain [11], Zimbabwe [62], and multiple other nations [50]. Interestingly, we noted in the current survey that a small proportion of participants reported increased time for physical activity and sleep. Two-thirds indicated no change during the COVID-19 period as compared to the pre-COVID-19. It is unclear what factors enabled most participants in our survey to maintain physical activity and sleep habits. This neutral observation on physical activity may be attributed to different regional governmental policies and socioeconomic factors anticipated to affect individuals’ lifestyle behaviors [56,59]. Moreover, some individuals may increase engagement in physical activity by taking advantage of home confinement.

There are a few limitations to this study. As the data were self-reported by participants, there may be the potential presence of recall bias, underreporting, and inaccuracy, which are all inherent limitations of online survey studies. Nevertheless, it has been reported that the data quality of web-based surveys was comparable to conventional face-to-face interviews [63]. The study findings might not be generalizable to the whole population in India because the study participants were limited to those in the four largest cities in India and in SEC A and B to capture urban dwellers who are active internet users (critical for completion of the online survey); they were also selected based on age (18–50 years), occupation, and household structure. Participants were also mostly highly educated (78% achieved a graduate/post-graduate education level). Additionally, the findings are not generalizable to other countries due to differences in lifestyle and dietary behaviors. Categorical data of self-reported BW changes limited our capability to assess whether the changes were clinically significant. Given the cross-sectional nature of the data, findings based on the data gathered in 2020 at the start of the pandemic may not represent perceived health and self-reported BW changes and lifestyle habits further on in the pandemic. Additionally, it is not possible to predict how the changes in diet and lifestyle behaviors observed during the COVID-19 period would evolve as the pandemic progresses and following the end of the pandemic. Nevertheless, the information collected in this study may help inform the formulation of strategies for lifestyle habits supporting health and well-being post COVID-19 and during future pandemics.

## 5. Conclusions

In this online survey conducted in India in early 2021, we found that the overall perceived health was not altered but improved in most participants during the COVID-19 pandemic. Additionally, an increase in BW was self-reported by most participants. The major health concerns among participants included mental health, BW management, low energy, lack of immunity, and digestive issues during the COVID-19 pandemic. Not surprisingly, both negative and positive changes in lifestyle and dietary habits were reported by the survey participants, such as binge eating and consuming the main meals at later times for the negative and eating healthier foods, including oats, millets, fruits, and plain dal/sambhar, and home-cooked foods, increasing time for physical activity, sleep, cooking, and relaxation activity, and tracking food consumption for the positive. The top reasons for improving dietary habits included improving physical and mental health and building immunity. The changes in lifestyle and dietary habits may have a long-lasting effect on health, immunity, and BW, thus, education on nutrition and food choices, such as whole grains, fruit/vegetables, and micronutrients could help support health, BW, and immunity.

## Figures and Tables

**Table 1:** Characteristics of study participants.

		*n*	%
Gender	Female	500	50.0
	Male	500	50.0
Generation	Gen X (41–50 y/o)	100	10.0
	Millennial (26–40 y/o)	450	45.0
	Gen Z (18–25 y/o)	450	45.0
City	Bangalore	250	25.0
	Delhi/NCR	250	25.0
	Kolkata	250	25.0
	Mumbai	250	25.0
Education level of chief wage earner	Graduate/Postgraduate: Professional	308	30.8
	Graduate/Postgraduate: General	469	46.9
	Some College (including Diploma) but not Grad	122	12.2
	SSC/HSC	59	5.9
	School—5–9 years	18	1.8
	Literate but no formal schooling/school—up to 4 years	8	0.8
	Illiterate	16	1.6

Abbreviations: Gen—generation; SSC/HSC—secondary school certificate/high school certificate; y/o—years old.

**Table 2:** The change in self-reported intake frequency of 14 food groups during the COVID-19 confinement compared to pre-COVID-19 *.

Food Group	Pre-COVID-19 Frequency	during-COVID-19 Frequency			Test of Symmetry	
		<3 Times a Week	4–5 Times a Week to Twice a Day	≥3 Times a Day	Unadjusted *p*-Value	Adjusted *q*-Value
Cooked brown/red/black rice	<3 times a week	225 (22.5)	67 (6.7)	31 (3.1)	0.24	0.37
	From 4–5 times a week to twice a day	57 (5.7)	394 (39.4)	67 (6.7)		
	≥3 times a day	22 (2.2)	52 (5.2)	85 (8.5)		
Cooked leafy green and other vegetables	<3 times a week	38 (3.8)	39 (3.9)	12 (1.2)	0.32	0.40
	From 4–5 times a week to twice a day	36 (3.6)	567 (56.7)	82 (8.2)		
	≥3 times a day	16 (1.6)	105 (10.5)	105 (10.5)		
Cooked millets	<3 times a week	236 (23.6)	77 (7.7)	20 (2.0)	0.046	0.13
	From 4–5 times a week to twice a day	48 (4.8)	390 (39.0)	69 (6.9)		
	≥3 times a day	26 (2.6)	61 (6.1)	73 (7.3)		
Cooked oats	<3 times a week	206 (20.6)	58 (5.8)	24 (2.4)	0.94	0.94
	From 4–5 times a week to twice a day	59 (5.9)	405 (40.5)	67 (6.7)		
	≥3 times a day	20 (2.0)	65 (6.5)	96 (9.6)		
Eggs	<3 times a week	175 (17.5)	46 (4.6)	21 (2.1)	0.28	0.39
	From 4–5 times a week to twice a day	50 (5.0)	493 (49.3)	77 (7.7)		
	≥3 times a day	11 (1.1)	68 (6.8)	59 (5.9)		
Fruits	<3 times a week	54 (5.4)	41 (4.1)	13 (1.3)	0.024	0.11
	From 4–5 times a week to twice a day	37 (3.7)	584 (58.4)	102 (10.2)		
	≥3 times a day	20 (2.0)	66 (6.6)	83 (8.3)		
Meat and seafood	<3 times a week	324 (32.4)	70 (7.0)	26 (2.6)	0.042	0.13
	From 4–5 times a week to twice a day	44 (4.4)	367 (36.7)	46 (4.6)		
	≥3 times a day	17 (1.7)	52 (5.2)	54 (5.4)		
Milk/curd/paneer (dairy)	<3 times a week	73 (7.3)	38 (3.8)	15 (1.5)	0.65	0.73
	From 4–5 times a week to twice a day	48 (4.8)	577 (57.7)	71 (7.1)		
	≥3 times a day	19 (1.9)	70 (7.0)	89 (8.9)		
Other milk products (cheese, khoa)	<3 times a week	212 (21.2)	55 (5.5)	16 (1.6)	0.15	0.26
	From 4–5 times a week to twice a day	62 (6.2)	414 (41.4)	88 (8.8)		
	≥3 times a day	16 (1.6)	61 (6.1)	76 (7.6)		
Plain dal/sambhar	<3 times a week	87 (8.7)	53 (5.3)	20 (2.0)	0.002	0.023
	From 4–5 times a week to twice a day	31 (3.1)	610 (61.0)	68 (6.8)		
	≥3 times a day	10 (1.0)	42 (4.2)	79 (7.9)		
Vegetables—salads	<3 times a week	78 (7.8)	47 (4.7)	25 (2.5)	0.67	0.73
	From 4–5 times a week to twice a day	36 (3.6)	491 (49.1)	94 (9.4)		
	≥3 times a day	27 (2.7)	94 (9.4)	108 (10.8)		
White flour	<3 times a week	263 (26.3)	70 (7.0)	24 (2.4)	0.10	0.24
	From 4–5 times a week to twice a day	46 (4.6)	370 (37.0)	66 (6.6)		
	≥3 times a day	25 (2.5)	54 (5.4)	82 (8.2)		
White rice	<3 times a week	80 (8.0)	44 (4.4)	19 (1.9)	0.007	0.048
	From 4–5 times a week to twice a day	30 (3.0)	623 (62.3)	77 (7.7)		
	≥3 times a day	10 (1.0)	48 (4.8)	69 (6.9)		
Whole wheat	<3 times a week	69 (6.9)	29 (2.9)	13 (1.3)	0.13	0.26
	From 4–5 times a week to twice a day	26 (2.6)	606 (60.6)	67 (6.7)		
	≥3 times a day	26 (2.6)	80 (8.0)	84 (8.4)		

* Bowker’s test of symmetry was performed to compare self-reported dietary intake pre- and during-COVID-19. A false discovery rate (FDR) adjustment was applied for multiple comparisons. A FDR (*q*-value) < 0.05 was considered significant.

**Table 3:** The change in self-reported lifestyle behaviors during the COVID-19 confinement compared to pre-COVID-19 *.

Lifestyle Parameter	Pre-COVID-19 Time	during-COVID-19 Time			Test of Symmetry	
		<30 min	from 30 min to 3 h	>3 h	Unadjusted *p*-Value	Adjusted *q*-Value
Physically active	<30 min	132 (13.2)	122 (12.2)	12 (1.2)	<0.001	<0.001
	From 30 min to 3 h	79 (7.9)	449 (44.9)	78 (7.8)		
	>3 h	5 (0.5)	48 (4.8)	75 (7.5)		
		<5 h	6–8 h	>8 h		
Sleeping	<5 h	274 (27.4)	74 (7.4)	26 (2.6)	<0.001	<0.001
	6–8 h	68 (6.8)	316 (31.6)	133 (13.3)		
	>8 h	7 (0.7)	24 (2.4)	78 (7.8)		
		<1 h	1–5 h	≥6 h		
Cleaning	<1 h	388 (38.8)	185 (18.5)	13 (1.3)	<0.001	<0.001
	1–5 h	88 (8.8)	230 (23.0)	29 (2.9)		
	≥6 h	14 (1.4)	25 (2.5)	28 (2.8)		
Connecting virtually	<1 h	314 (31.4)	201 (20.1)	13 (1.3)	<0.001	<0.001
	1–5 h	98 (9.8)	254 (25.4)	45 (4.5)		
	≥6 h	6 (0.6)	27 (2.7)	42 (4.2)		
Cooking	<1 h	348 (34.8)	194 (19.4)	12 (1.2)	<0.001	<0.001
	1–5 h	90 (9.0)	263 (26.3)	38 (3.8)		
	≥6 h	8 (0.8)	20 (2.0)	27 (2.7)		
Doing professional work	<1 h	132 (13.2)	75 (7.5)	9 (0.9)	<0.001	<0.001
	1–5 h	81 (8.1)	300 (30.0)	60 (6.0)		
	≥6 h	31 (3.1)	122 (12.2)	190 (19.0)		
Leisure entertainment activities	<1 h	217 (21.7)	188 (18.8)	15 (1.5)	<0.001	<0.001
	1–5 h	92 (9.2)	344 (34.4)	54 (5.4)		
	≥6 h	9 (0.9)	36 (3.6)	45 (4.5)		
On social media	<1 h	236 (23.6)	185 (18.5)	10 (1.0)	<0.001	<0.001
	1–5 h	89 (8.9)	346 (34.6)	43 (4.3)		
	≥6 h	10 (1.0)	32 (3.2)	49 (4.9)		
Socializing with family or roommates	<1 h	281 (28.1)	134 (13.4)	11 (1.1)	0.93	0.93
	1–5 h	134 (13.4)	286 (28.6)	45 (4.5)		
	≥6 h	11 (1.1)	39 (3.9)	59 (5.9)		
Travelling in the city	<1 h	350 (35.0)	91 (9.1)	7 (0.7)	<0.001	<0.001
	1–5 h	269 (26.9)	176 (17.6)	24 (2.4)		
	≥6 h	31 (3.1)	24 (2.4)	28 (2.8)		
Watching recipe related videos	<1 h	358 (35.8)	190 (19.0)	20 (2.0)	<0.001	<0.001
	1–5 h	89 (8.9)	238 (23.8)	42 (4.2)		
	≥6 h	5 (0.5)	18 (1.8)	40 (4.0)		

* Bowker’s test of symmetry was performed to compare self-reported lifestyle behaviors pre- and during-COVID confinement. A false discovery rate (FDR) adjustment was applied for multiple comparisons. FDR (*q*-value) < 0.05 was considered significant.

## Data Availability

The data presented in this study are available on request from the corresponding author. The data are not publicly available due to proprietary reasons.

## Supplementary Materials

The following are available online at https://www.mdpi.com/article/10.3390/nu13113682/s1, Supplemental Survey Questions S1; Table S1: Concerns about health conditions considering health at the time of survey; Supplemental Table S2: Diet regimens used to manage body weight in the subset of participants reporting a concern for body weight during the COVID-19 confinement; Supplemental Table S3: Change in the intake frequency of 14 food groups during the COVID-19 confinement as compared to pre-COVID-19; Supplemental Table S4: Regimens intended to improve or maintain physical and mental health started during the COVID-19 confinement; Supplemental Table S5: Association between dietary intake changes and (1) city, (2) generation, (3) perceived health change, (4) gender, and (5) self-reported body weight.
