# Supplementary material for: Effect of COVID-19 Pandemic-Induced Dietary and Lifestyle Changes and Their Associations with Perceived Health Status and Self-Reported Body Weight Changes in India: A Cross-Sectional Survey
Source: Nutrients. 2021 Oct 20;13(11):3682. doi: 10.3390/nu13113682 (PMC8620355; doi:10.3390/nu13113682)
Supplement: Supplementary file 1 [file nutrients-13-03682-s001.zip › Madan et al - Supplemental tables - 17Sep2021.pdf]

Supplementary Tables

Supplementary Table S1. Concerns about health conditions considering health at the time of survey *.

	n	%
Concerned about digestive issues		
Unconcerned	114	11.40
Neutral	200	20.00
Concerned	686	68.60
Concerned about lack of immunity		
Unconcerned	132	13.20
Neutral	172	17.20
Concerned	696	69.60
Concerned about obesity/weight management		
Unconcerned	120	12.00
Neutral	168	16.80
Concerned	712	71.20
Concerned about diabetes/sugar/glucose control		
Unconcerned	190	19.00
Neutral	198	19.80
Concerned	612	61.20
Concerned about cholesterol		
Unconcerned	158	15.80
Neutral	207	20.70
Concerned	635	63.50
Concerned about hypertension/blood pressure		
Unconcerned	165	16.50
Neutral	202	20.20
Concerned	633	63.30

	n	*%*
Concerned about virtual fatigue		
Unconcerned	139	13.90
Neutral	201	20.10
Concerned	660	66.00
Concerned about fatigue		
Unconcerned	119	11.90
Neutral	185	18.50
Concerned	696	69.60
Concerned about joint and muscle pain		
Unconcerned	159	15.90
Neutral	209	20.90
Concerned	632	63.20
Concerned about health health and heart disease		
Unconcerned	155	15.50
Neutral	203	20.30
Concerned	642	64.20
Concerned about stress and anxiety		
Unconcerned	110	11.00
Neutral	168	16.80
Concerned	722	72.20
Concerned about depression and mental health		
Unconcerned	119	11.90
Neutral	183	18.30
Concerned	698	69.80
Concerned about sleeping problems		
Unconcerned	147	14.70

	n	%
Neutral	201	20.10
Concerned	652	65.20
Concerned about women's health	46	9.20
Unconcerned		
Neutral	90	18.00
Concerned	364	72.80

*The table summarizes percentage of subjects reporting no concern (response options unconcerned and not at all concerned), neutral, or concern (response options concerned and very concerned) regarding queried health conditions. Concern about women's health was only asked to women.

Supplementary Table S2. Diet regimens used to manage body weight in the subset of subjects reporting a concern for body weight during the COVID-19 confinement.

	N	%
Keto diet		
Yes	235	33.01
No	477	66.99
Intermittant fasting		
Yes	260	36.52
No	452	63.48
Low carbohydrate/high protein		
Yes	310	43.54
No	402	56.46
Customized diet plans by nutritionists		
Yes	199	27.95
No	513	72.05
GM diet		
Yes	176	24.72
No	536	75.28
Weekly detoxing plan		
Yes	185	25.98
No	527	74.02
Liquid diet		
Yes	164	23.03
No	548	76.97
Home-made cooked food only		
Yes	339	47.61
No	373	52.39

	N	*%*
None of the listed diets		
Yes	27	3.79
No	685	96.21

Supplementary Table S3. Change in the intake frequency of 14 food groups during the COVID-19 confinement as compared to pre-COVID-19.

		n	%
Cooked leafy green and other vegetables	*Decrease*	290	29.00
	No change	476	47.60
	Increase	234	23.40
Plain dal/sambhar	*Decrease*	194	19.40
	No change	571	57.10
	Increase	235	23.50
Eggs	*Decrease*	217	21.70
	No change	524	52.40
	Increase	259	25.90
Fruits	*Decrease*	241	24.10
	No change	441	44.10
	Increase	318	31.80
Meat and seafood	*Decrease*	207	20.70
	No change	548	54.80
	Increase	245	24.50
Other milk products (cheese, khoa)	*Decrease*	245	24.50
	No change	487	48.70
	Increase	268	26.80
Milk/curd/paneer (dairy)	*Decrease*	247	24.70
	No change	503	50.30
	Increase	250	25.00
Cooked millets	*Decrease*	240	24.00
	No change	487	48.70
	Increase	273	27.30

		n	%
Cooked oats	*Decrease*	237	23.70
	No change	491	49.10
	Increase	272	27.20
Cooked brown/red/black rice	*Decrease*	248	24.80
	No change	489	48.90
	Increase	263	26.30
Vegetables - salads	*Decrease*	249	24.90
	No change	454	45.40
	Increase	297	29.70
White flour	*Decrease*	217	21.70
	No change	525	52.50
	Increase	258	25.80
Whole wheat	*Decrease*	238	23.80
	No change	566	56.60
	Increase	196	19.60
White rice	*Decrease*	174	17.40
	No change	591	59.10
	Increase	235	23.50

Supplementary Table S4. Regimens intended to improve or maintain physical and mental health started during the COVID confinement.

	n	%
Aerobics/Cardio/dance fitness		
Yes	286	28.60
No	714	71.40
Yoga practice		
Yes	578	57.80
No	422	42.20
Breathing exercises		
Yes	518	51.80
No	482	48.20
Walking/running		
Yes	557	55.70
No	443	44.30
Meditation		
Yes	430	43.00
No	570	57.00
Strength training		
Yes	278	27.80
No	722	72.20
Other physical/mental health regimens		
Yes	8	0.80
No	992	99.20
Haven't started any physical/mental activities		
Yes	59	5.90
No	941	94.10

Supplementary Table S5. Association between dietary intake changes and (1) city, (2) generation, (3) perceived health change, (4) gender, and (5) self-reported body weight.

Factor	*Diet change*	*Unadjusted p-value*	*Adjusted FDR q-value*
City	Fruits	0.002	0.020
	White rice	0.006	0.044
	Plain dal/sambhar	0.009	0.056
	Whole wheat	0.216	0.378
	Eggs	0.275	0.459
	Vegetables - salads	0.348	0.511
	Other milk products (cheese, khoa)	0.359	0.514
	Cooked leafy green and other vegetables	0.500	0.625
	Cooked oats	0.630	0.735
	Cooked brown/red/black rice	0.665	0.764
	White flour	0.716	0.795
	Milk/curd/paneer (dairy)	0.799	0.847
	Cooked millets	0.823	0.859
	Meat and seafood	0.880	0.893
Generation	Cooked oats	0.002	0.020
	Plain dal/sambhar	0.008	0.054
	Eggs	0.036	0.126
	Fruits	0.069	0.180
	White rice	0.119	0.261
	Cooked leafy green and other vegetables	0.130	0.276
	Meat and seafood	0.139	0.287
	Cooked millets	0.175	0.326
	Vegetables - salads	0.177	0.326
	Cooked brown/red/black rice	0.207	0.371
	Other milk products (cheese, khoa)	0.312	0.481

Factor	*Diet change*	*Unadjusted p-value*	*Adjusted FDR q-value*
	Whole wheat	0.464	0.625
	Milk/curd/paneer (dairy)	0.485	0.625
	White flour	0.786	0.846
Health change	Cooked oats	<0.001	0.005
	Milk/curd/paneer (dairy)	0.010	0.056
	Cooked brown/red/black rice	0.018	0.079
	Fruits	0.028	0.108
	White rice	0.031	0.115
	Whole wheat	0.045	0.128
	Vegetables - salads	0.046	0.128
	Meat and seafood	0.046	0.128
	Eggs	0.102	0.239
	Plain dal/sambhar	0.169	0.326
	Cooked millets	0.316	0.481
	White flour	0.316	0.481
	Other milk products (cheese, khoa)	0.316	0.481
	Cooked leafy green and other vegetables	0.596	0.707
Gender	Meat and seafood	0.064	0.174
	Vegetables - salads	0.116	0.261
	White rice	0.248	0.423
	Cooked brown/red/black rice	0.350	0.511
	Fruits	0.385	0.539
	Plain dal/sambhar	0.475	0.625
	Cooked millets	0.481	0.625
	Whole wheat	0.500	0.625
	Milk/curd/paneer (dairy)	0.518	0.636

Factor	*Diet change*	*Unadjusted p-value*	*Adjusted FDR q-value*
	Eggs	0.585	0.706
	Cooked leafy green and other vegetables	0.685	0.774
	Cooked oats	0.763	0.835
	White flour	0.863	0.889
	Other milk products (cheese, khoa)	0.941	0.941
Weight change	Milk/curd/paneer (dairy)	<.001	0.008
	Cooked leafy green and other vegetables	<.001	0.008
	Cooked oats	<.001	0.008
	Vegetables - salads	0.002	0.020
	Meat and seafood	0.002	0.020
	Other milk products (cheese, khoa)	0.011	0.059
	Eggs	0.012	0.061
	Plain dal/sambhar	0.016	0.076
	Fruits	0.020	0.082
	White flour	0.043	0.128
	Cooked millets	0.044	0.128
	White rice	0.096	0.239
	Whole wheat	0.101	0.239
	Cooked brown/red/black rice	0.153	0.305

The association between the factor of interest (city, generation, gender, self-reported body weight change, and perceived health change) and the diet change was evaluated using the Chi square test. A false discovery rate (FDR) adjustment was applied for multiple comparisons.
