# Supplementary material for: Effect of COVID-19 Pandemic-Induced Dietary and Lifestyle Changes and Their Associations with Perceived Health Status and Self-Reported Body Weight Changes in India: A Cross-Sectional Survey
Source: Nutrients. 2021 Oct 20;13(11):3682. doi: 10.3390/nu13113682 (PMC8620355; doi:10.3390/nu13113682)
Supplement: Supplementary file 1 [file nutrients-13-03682-s001.zip › Madan et al - Survey questions - 17Sep2021.pdf]

Survey questions

Lifestyle Changes

Firstly, we would like to ask you a few questions about your daily routine in the pre-COVID period and during COVID period –

1. On a typical day in the Pre-COVID period, how much time did you use to spend on the following activities? By Pre-COVID, we mean before the first lockdown (March 2020)
2. On a typical day during COVID period, how much time have you been spending on the following activities? By during-COVID, we mean from April 2020 to present

For Q1 and Q2	Less than 30 mins	30 mins to 1 hour	1-3 hours	3-5 hours	6-8 hours	More than 8 hours	Not Applicable
Doing professional work (office/studies)							
Cooking food							
Cleaning and other domestic chores							
Participate in physical exercise							
Socialize with family or roommates who live with me							
Spend time on social media (Facebook, Instagram, Twitter, etc.)							

Connect with friends or family virtually on the phone, Skype, WhatsApp, etc.							
Leisure entertainment activities like watching TV, playing video games							
Travelling in the city (for work, shopping, college, etc.)							
Sleep							
Watching recipe related videos online/ on TV							

3. On a typical day in the Pre-COVID period, at what times would you eat the following meals? By meals, we mean all the meals in a day, including breakfast, lunch, dinner, and snacks

    Breakfast

    Lunch

    Dinner

    Any Snacks in the day

4. On a typical day during COVID period, at what times would you eat the following meals? By meals, we mean all the meals in a day, including breakfast, lunch, dinner, and snacks

    Breakfast

Lunch

Dinner

Any Snacks in the day

5. Thinking of a usual day during COVID months, which of the following best describes how you have your meals?

	Breakfast	Lunch	Dinner
With Family			
With doing office work			
While watching TV/Mobile			
While reading/listening music			

6. Thinking about your general habits during COVID period, are there any meals that you tend to skip more than others?

Breakfast

Snack between breakfast and lunch

Lunch

Snack between lunch and dinner

Dinner

Late-night snack (after dinner)

I don't skip any of these meals

7. How often do you skip breakfast in a week? [Only asked if Q6 1 was selected]

1 time a week

2 times a week

3 times a week

4 times a week

5 times a week

6 times a week

7 times a week (everyday)

8. Which of the following reasons best describes why you skip breakfast? [Only asked if Q6 1 was selected]

I don’t get time to eat breakfast in the morning

I start my day around noon due to my work schedule, so I wait till lunch

I don’t feel hungry enough in the morning

I don’t get time to cook a meal for breakfast

I don’t consider breakfast as an important meal of the day

Any other reason, please specify…….

9. What is the breakfast which you consume most often? [open ended]
10. Which of the following statements about breakfast do you agree with? [Select all that apply]

Skipping breakfast helps me lose weight

Skipping breakfast helps me maintain my calorie intake

A glass of milk or a fruit is enough to have for breakfast

Breakfast should be rich in protein and fibre

Making nutritious and tasty breakfast takes a lot of time

Breakfast should be low in carbohydrates

11. How does breakfast help you through the day? [Select all that apply]

Makes me feel fuller for longer

Give energy for at least half a day

Stops me from binge eating unhealthier food options

Others, please specify…….

I don't think breakfast is an important meal

12. What is your favourite breakfast? [open ended]

Dietary Intake

13. Thinking about the Pre-COVID period (*By Pre-COVID, we mean before the first lockdown (March 2020),* please tell us how often you consume the following foods *(please think about all the meals in the day while answering – breakfast, lunch, dinner and snack).*
14. Now, please think about the during COVID period (*By during-COVID, we mean April-2020 to present)*, please tell us how often you consume the following foods *(please think about all the meals in the day while answering – breakfast, lunch, dinner and snack).*

**Food Group**	**Frequency***	**Serving per Occasion**
Fruits (medium-size)		Number (1 fruit/100 grams)
Cooked green leafy and other vegetables – spinach, methi, cabbage, cauliflower, ladyfingers, etc (gravy/dry)		Cups (200 grams)
Cooked roots and tubers – potatoes, carrot, beetroot, etc (gravy/dry)		Cups (200 grams)
Vegetables - Salads		1-4 of slices (10 grams)
Eggs (boiled/omelette)		Number
Chicken/mutton/fish/prawn pieces (in Curry/Fried)		Cups (200 grams)
**Dairy**		
Milk/Curd/Paneer		Milk/Curd -Cups (200 ml)/Paneer (30 grams)
Other Milk products (Cheese, Khoa)		Tablespoon (15 grams)
**Wholegrains**		
Whole Wheat (Chapati/roti/)		Number of chapati/roti(30 grams)
Cooked Oats (upma, porridge, kichadi)		Cups (200 grams)
Cooked Millets (Ragi millet dosa/idli/roti/bakhri/Kichadi)		Number 2 idlis/1 dosa /1 roti/1/2 bhakri/1 cup khichdi (30 grams)

Cooked Brown/red/black rice (plain/pulav)		Cups (200 grams)
**Refined grains**		
Cooked White rice (plain/pulav)		Cups (200 grams)
White flour (maida) – Luchi, Naan, Kulcha		Number of Kulchas/luchi/Naan (30 grams)
**Pulses**		
Plain dal/Sambhar		Cups (200 grams)

**Frequency*

- *Once a day*
- *Twice a day*
- *Thrice a day*
- *4-5 times a day*
- *1-2 times a week*
- *2-3 times a week*
- *4-5 times a week*
- *I don't eat it at all*

Health & Nutrition Status - Perception & Concerns

15. In general, how would you rate your current health? [Poor, fair, good, very good, excellent]
16. Has your health improved, deteriorated, or remained the same compared to the pre-COVID months? [improved, deteriorated, remained the same]
17. Has your weight increased, decreased, or remained the same during the COVID months (since lockdown April 2020-present)? [Increased, decreased, remained the same]
18. Considering your present health, could you please tell us how concerned you are about the following health conditions?

	Very Concerned (5)	Concerned (4)	Neutral (3)	Unconcerned (2)	Not at all Concerned (1)
Digestive issues					
Lack of Immunity (frequent cough, cold, flu)					

Obesity/Weight management					
Diabetes/Sugar/Glucose Control					
Virtual Fatigue					
Physical Tiredness and Fatigue/Low Energy					
Joint and muscle pain					
Heart health and heart disease					
Stress and Anxiety					
Depression and mental health					
Sleeping problems					
Women's health *[only show if marked as Female in Gender]*					
Cholesterol issues					
Hypertension and Blood Pressure issues					

19. You mentioned that you are concerned about your weight; please tell us which of the following diet regimens have you tried to manage your weight during the COVID months? [asked if Q18 obesity/weight management marked as 4/5, select all that apply]

Keto diet

Intermittent fasting

Low carb, high protein diet

Customized diet plans by nutritionists

GM diet plan

Weekly detoxing plan

Liquid diet

Home-made cooked food only

Others, please specify

None of the above

20. Please tell us which of the following physical/mental health regimens have you started during the COVID months? [select all that apply]

Aerobics/Cardio/dance fitness

Yoga practice

Breathing exercises

Walking/running

Meditation

Strength training

Others, please specify

I haven't started any physical/mental activities

21. How much do you agree or disagree with the following statements about your habits during the COVID period?

	Strongly Agree (5)	Agree (4)	Neither Agree nor Disagree (3)	Disagree (2)	Strongly Disagree (1)
I feel that my nutrition intake has increased overall					

I have started eating more healthy food

I have started binge eating more

I have started eating more home-cooked food

I have started eating lot of snacks in between the meals

My portion size of a meal has increased

I keep a track of what and how much I eat in the day in terms of calories

I started cooking more often at home

I started looking for healthy recipes to cook at home

I feel discouraged to cook

22. You mentioned earlier that you changed some of your eating habits relating to certain foods. Which of the following factors drove you to change your eating habits during the COVID period? [Select all that apply]

Improve physical health

No time to cook

Don’t know how to cook

Convenience of ready-to-eat/cook food

Improve mental health (stress, anxiety)

Not over-eat during the day

Reduce weight

Build immunity

Any other reason, please specify…….

23. What all have you included in your diet to build immunity during the COVID period? Please think of any ingredient, food type, or packaged foods. [open ended]

Packaged food and choices

24. For which of the following packaged foods do you look for specific healthy ingredients (like oats, tumeric, tulsi, methi, green tea, etc.) while purchasing? [select all that apply]

Cookies and biscuits
Chips/wafers
Dry savory snacks
Ready to cook/eat mixes (idli, dosa, etc.)
Bakery products (breads, buns, croissants, rusk, etc.)
Noodles, Pasta, Spaghetti
Beverages (Tea, milkshakes, juices, etc.)
Breakfast cereals
Any other, please specify……………………………..

25. What are the factors which influence you to buy certain ingredients/products? Please select all that apply.

Medical recommendation

Nutritionist/dietician recommendation

Read or heard in product advertisement

Recommendation from social media

Recommendation from friends or family

Others, please specify………

26. Which new ingredients and/or packaged foods, if any, have you tried during the COVID period for any of your meals? [open ended]

Oats Consumption

27. What was your frequency of consuming oats (in any form) during the pre-COVID period?

Thrice a day or more

Twice a day

Once a day

Once in 2 days

Once in 3-4 days

Once in a week

Once in two weeks

Once a month

Less than once a month

Never consumed in pre-COVID months

28. Has your consumption of oats during COVID to support immunity period increased, decreased, or stayed the same? [increased, decreased, remained the same]
29. Why do you consume oats?

Helps manage weight

Rich in Fiber

Rich in proteins

Provides micronutrients that nutrients

Give long lasting energy

Help support heart related issues

Is Nutritious and healthy

Breakfast I look forward to

Is easy to prepare / convenient to cook

Easy to digest

Helps strengthen immunity

It is natural / Is made with natural ingredients

Is recommended by fitness trainer/gym instructor/sports coach

Is recommended by Doctor/Dietician/Nutritionist

Others, please specify………………………………………….

30. Generally, when do you consume oats in a typical day? [breakfast, lunch, dinner, snacks, select all that apply]
31. How do you like to consume oats most often? [as a main dish, as an ingredient in the main dish]
32. Generally, in what form (recipe) do you like to consume oats? [select all that apply]
    - smoothie
    - khichdi
    - oats upma/dosa
    - oats chilla
    - oats patties
    - oats idlis
    - other, specify

Purchase Intent

33. Thinking about your general grocery purchases, which medium have you been using more often during COVID period? *By during-COVID, we mean since lockdown (April 2020-present) [single select response]*
    - Mostly go to offline stores (like local shops, grocery stores, supermarkets, malls) for purchases
    - Mostly ordering from Offline stores (like local shops, grocery stores, supermarkets, malls) and getting home delivered
    - Mostly Online purchasing (from e-commerce websites like Amazon, Big Basket, Grofers, etc.)
34. Are you willing to pay a little more to purchase healthy food products or ingredients? [yes, no, not sure/can't say]
