# Supplementary material for: Effect of COVID-19 Pandemic-Induced Dietary and Lifestyle Changes and Their Associations with Perceived Health Status and Self-Reported Body Weight Changes in India: A Cross-Sectional Survey
Source: Nutrients. 2021 Oct 20;13(11):3682. doi: 10.3390/nu13113682 (PMC8620355; doi:10.3390/nu13113682)
Supplement: Supplementary file 1 [file nutrients-13-03682-s001.zip › Madan et al - Tables and Figures_17Sep2021.pdf]

Table S1. Characteristics of study participants.

		N	%
Gender	Female	500	50.0
	Male	500	50.0
Generation	Gen X	100	10.0
	Millennial	450	45.0
	Gen Z	450	45.0
City	Bangalore	250	25.0
	Delhi/NCR	250	25.0
	Kolkata	250	25.0
	Mumbai	250	25.0
Education level of chief wage earner	Graduate/ Post Graduate: Professional	308	30.8
	Graduate/ Post Graduate: General	469	46.9
	Some College (including Diploma) but not Grad	122	12.2
	SSC/ HSC	59	5.9
	School-5 to 9 years	18	1.8
	Literate but no formal schooling/ School-Up to 4 years	8	0.8
	Illiterate	16	1.6

Abbreviations: Gen = generation; SSC/HSC = secondary school certificate/high school certificate

Table S2. The change in self-reported intake frequency of 14 food groups during the COVID-19 confinement compared to pre-COVID-19 ***.**

Food Group	Pre-COVID Frequency	During COVID Frequency			Test of Symmetry	
		<3 times a week	*4-5 times a week to twice a day*	*≥3 times a day*	Unadjusted p-value	Adjusted q-value
Cooked brown/red/black rice	<3 times a week	225 (22.5)	67 (6.7)	31 (3.1)	0.24	0.37
	4-5 times a week to twice a day	57 (5.7)	394 (39.4)	67 (6.7)		
	≥3 times a day	22 (2.2)	52 (5.2)	85 (8.5)		
Cooked leafy green and other vegetables	<3 times a week	38 (3.8)	39 (3.9)	12 (1.2)	0.32	0.40
	4-5 times a week to twice a day	36 (3.6)	567 (56.7)	82 (8.2)		
	≥3 times a day	16 (1.6)	105 (10.5)	105 (10.5)		
Cooked millets	<3 times a week	236 (23.6)	77 (7.7)	20 (2.0)	0.046	0.13
	4-5 times a week to twice a day	48 (4.8)	390 (39.0)	69 (6.9)		
	≥3 times a day	26 (2.6)	61 (6.1)	73 (7.3)		
Cooked oats	<3 times a week	206 (20.6)	58 (5.8)	24 (2.4)	0.94	0.94
	4-5 times a week to twice a day	59 (5.9)	405 (40.5)	67 (6.7)		

	≥3 times a day	20 (2.0)	65 (6.5)	96 (9.6)		
Eggs	<3 times a week	175 (17.5)	46 (4.6)	21 (2.1)	0.28	0.39
	4-5 times a week to twice a day	50 (5.0)	493 (49.3)	77 (7.7)		
	≥3 times a day	11 (1.1)	68 (6.8)	59 (5.9)		
Fruits	<3 times a week	54 (5.4)	41 (4.1)	13 (1.3)	0.024	0.11
	4-5 times a week to twice a day	37 (3.7)	584 (58.4)	102 (10.2)		
	≥3 times a day	20 (2.0)	66 (6.6)	83 (8.3)		
Meat and seafood	<3 times a week	324 (32.4)	70 (7.0)	26 (2.6)	0.042	0.13
	4-5 times a week to twice a day	44 (4.4)	367 (36.7)	46 (4.6)		
	≥3 times a day	17 (1.7)	52 (5.2)	54 (5.4)		
Milk/curd/paneer (dairy)	<3 times a week	73 (7.3)	38 (3.8)	15 (1.5)	0.65	0.73
	4-5 times a week to twice a day	48 (4.8)	577 (57.7)	71 (7.1)		
	≥3 times a day	19 (1.9)	70 (7.0)	89 (8.9)		
Other milk products (cheese, khoa)	<3 times a week	212 (21.2)	55 (5.5)	16 (1.6)	0.15	0.26
	4-5 times a week to twice a day	62 (6.2)	414 (41.4)	88 (8.8)		
	≥3 times a day	16 (1.6)	61 (6.1)	76 (7.6)		
Plain dal/sambhar	<3 times a week	87 (8.7)	53 (5.3)	20 (2.0)	0.002	0.023

	4-5 times a week to twice a day	31 (3.1)	610 (61.0)	68 (6.8)		
	≥3 times a day	10 (1.0)	42 (4.2)	79 (7.9)		
Vegetables - salads	<3 times a week	78 (7.8)	47 (4.7)	25 (2.5)	0.67	0.73
	4-5 times a week to twice a day	36 (3.6)	491 (49.1)	94 (9.4)		
	≥3 times a day	27 (2.7)	94 (9.4)	108 (10.8)		
White flour	<3 times a week	263 (26.3)	70 (7.0)	24 (2.4)	0.10	0.24
	4-5 times a week to twice a day	46 (4.6)	370 (37.0)	66 (6.6)		
	≥3 times a day	25 (2.5)	54 (5.4)	82 (8.2)		
White rice	<3 times a week	80 (8.0)	44 (4.4)	19 (1.9)	0.007	0.048
	4-5 times a week to twice a day	30 (3.0)	623 (62.3)	77 (7.7)		
	≥3 times a day	10 (1.0)	48 (4.8)	69 (6.9)		
Whole wheat	<3 times a week	69 (6.9)	29 (2.9)	13 (1.3)	0.13	0.26
	4-5 times a week to twice a day	26 (2.6)	606 (60.6)	67 (6.7)		
	≥3 times a day	26 (2.6)	80 (8.0)	84 (8.4)		

*Bowker's test of symmetry was performed to compare self-reported dietary intake pre- and during-COVID-19. A false discovery rate (FDR) adjustment was applied for multiple comparisons. A FDR (q-value) <0.05 was considered significant.

Table S3. The change in self-reported lifestyle behaviors during the COVID-19 confinement compared to pre-COVID-19 ***.**

**Lifestyle Parameter**	**Pre-COVID Time**	**During COVID Time**			**Test of Symmetry**	
		<30 minutes	*30 minutes to 3 hours*	*> 3 hours*	Unadjusted p-value	Adjusted q-value
Physically active	*< 30 minutes*	132 (13.2)	122 (12.2)	12 (1.2)	<.001	<.001
	30 minutes to 3 hours	79 (7.9)	449 (44.9)	78 (7.8)		
	> 3 hours	5 (0.5)	48 (4.8)	75 (7.5)		
		< 5 hours	*6-8 hours*	*> 8 hours*		
Sleeping	*< 5 hours*	274 (27.4)	74 (7.4)	26 (2.6)	<.001	<.001
	6-8 hours	68 (6.8)	316 (31.6)	133 (13.3)		
	> 8 hours	7 (0.7)	24 (2.4)	78 (7.8)		
		< 1 hour	*1-5 hours*	*≥ 6 hours*		
Cleaning	*< 1 hour*	388 (38.8)	185 (18.5)	13 (1.3)	<.001	<.001
	1-5 hours	88 (8.8)	230 (23.0)	29 (2.9)		
	≥ 6 hours	14 (1.4)	25 (2.5)	28 (2.8)		
Connecting virtually	*< 1 hour*	314 (31.4)	201 (20.1)	13 (1.3)	<.001	<.001
	1-5 hours	98 (9.8)	254 (25.4)	45 (4.5)		
	≥ 6 hours	6 (0.6)	27 (2.7)	42 (4.2)		
Cooking	*< 1 hour*	348 (34.8)	194 (19.4)	12 (1.2)	<.001	<.001
	1-5 hours	90 (9.0)	263 (26.3)	38 (3.8)		
	≥ 6 hours	8 (0.8)	20 (2.0)	27 (2.7)		
Doing professional work	*< 1 hour*	132 (13.2)	75 (7.5)	9 (0.9)	<.001	<.001
	1-5 hours	81 (8.1)	300 (30.0)	60 (6.0)		
	≥ 6 hours	31 (3.1)	122 (12.2)	190 (19.0)		

Leisure entertainment activities	*< 1 hour*	217 (21.7)	188 (18.8)	15 (1.5)	<.001	<.001
	1-5 hours	92 (9.2)	344 (34.4)	54 (5.4)		
	≥ 6 hours	9 (0.9)	36 (3.6)	45 (4.5)		
On social media	*< 1 hour*	236 (23.6)	185 (18.5)	10 (1.0)	<.001	<.001
	1-5 hours	89 (8.9)	346 (34.6)	43 (4.3)		
	≥ 6 hours	10 (1.0)	32 (3.2)	49 (4.9)		
Socializing with family or roommates	*< 1 hour*	281 (28.1)	134 (13.4)	11 (1.1)	0.93	0.93
	1-5 hours	134 (13.4)	286 (28.6)	45 (4.5)		
	≥ 6 hours	11 (1.1)	39 (3.9)	59 (5.9)		
Travelling in the city	*< 1 hour*	350 (35.0)	91 (9.1)	7 (0.7)	<.001	<.001
	1-5 hours	269 (26.9)	176 (17.6)	24 (2.4)		
	≥ 6 hours	31 (3.1)	24 (2.4)	28 (2.8)		
Watching recipe related videos	*< 1 hour*	358 (35.8)	190 (19.0)	20 (2.0)	<.001	<.001
	1-5 hours	89 (8.9)	238 (23.8)	42 (4.2)		
	≥ 6 hours	5 (0.5)	18 (1.8)	40 (4.0)		

*Bowker's test of symmetry was performed to compare self-reported lifestyle behaviors pre- and during-COVID confinement. A false discovery rate (FDR) adjustment was applied for multiple comparisons. A FDR (q-value) < 0.05 was considered significant.

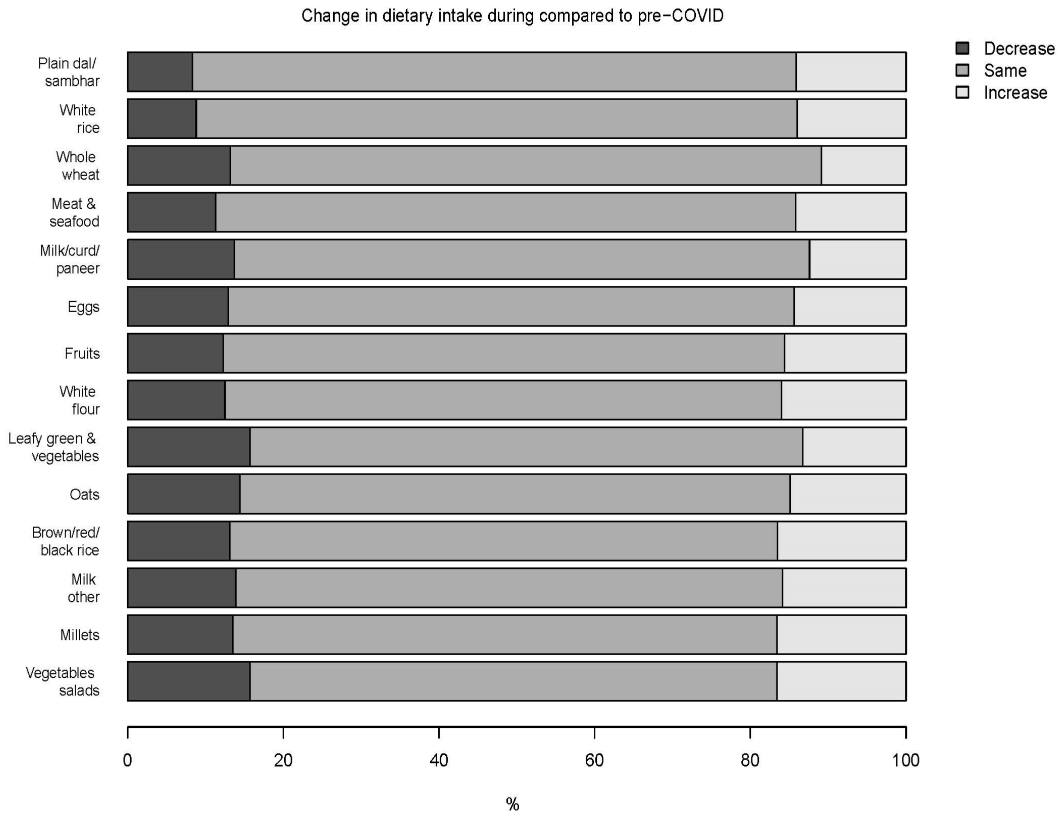

Figure S1. Percentage of participants reporting a decrease, no change (same), or increase in intake frequencies of 14 food groups during the COVID-19 confinement compared to pre-COVID-19.

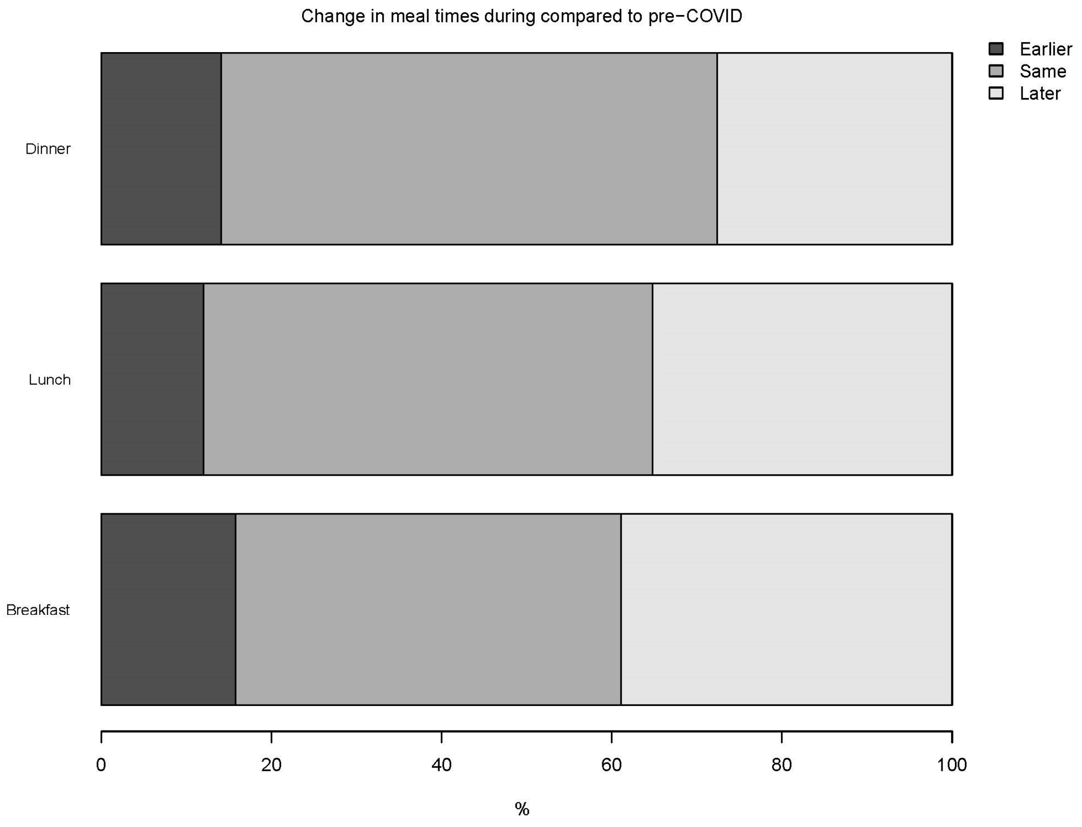

Figure S2. The percentage of respondents' change in meal times for breakfast, lunch, and dinner during the COVID-19 confinement compared to pre-COVID-19.

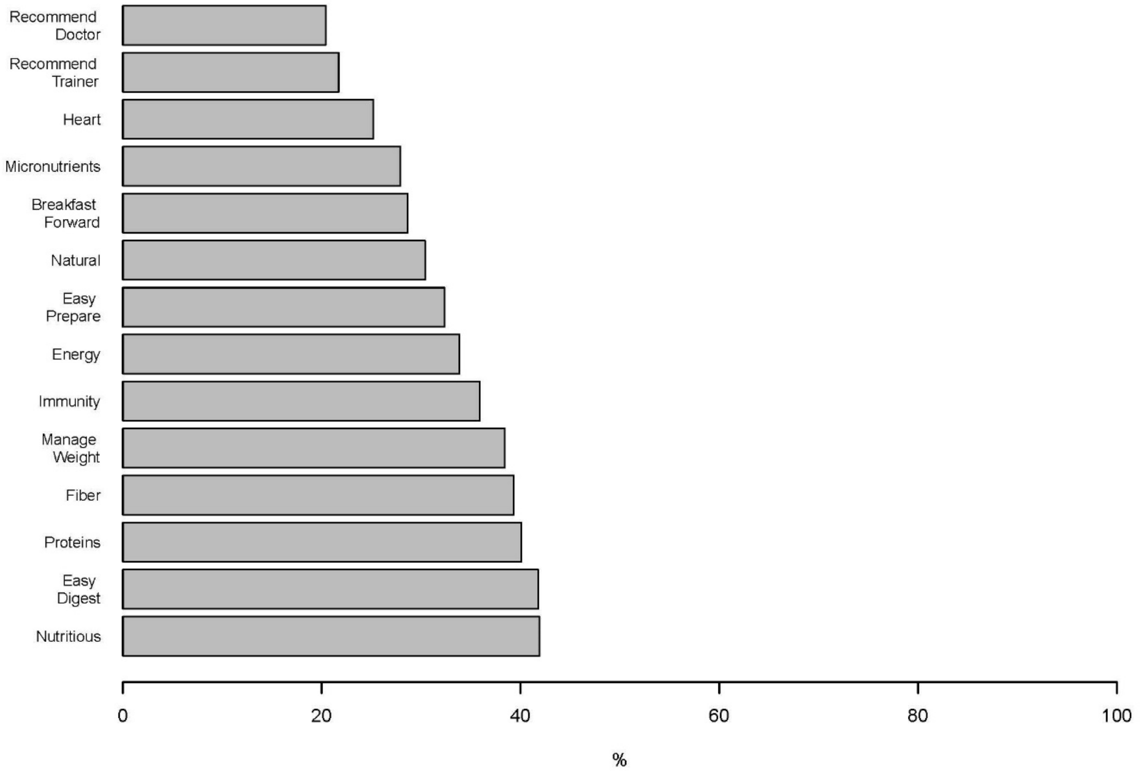

Figure S3. Top factors reported by percentage of participants contributing to oat consumption during the COVID-19 confinement.

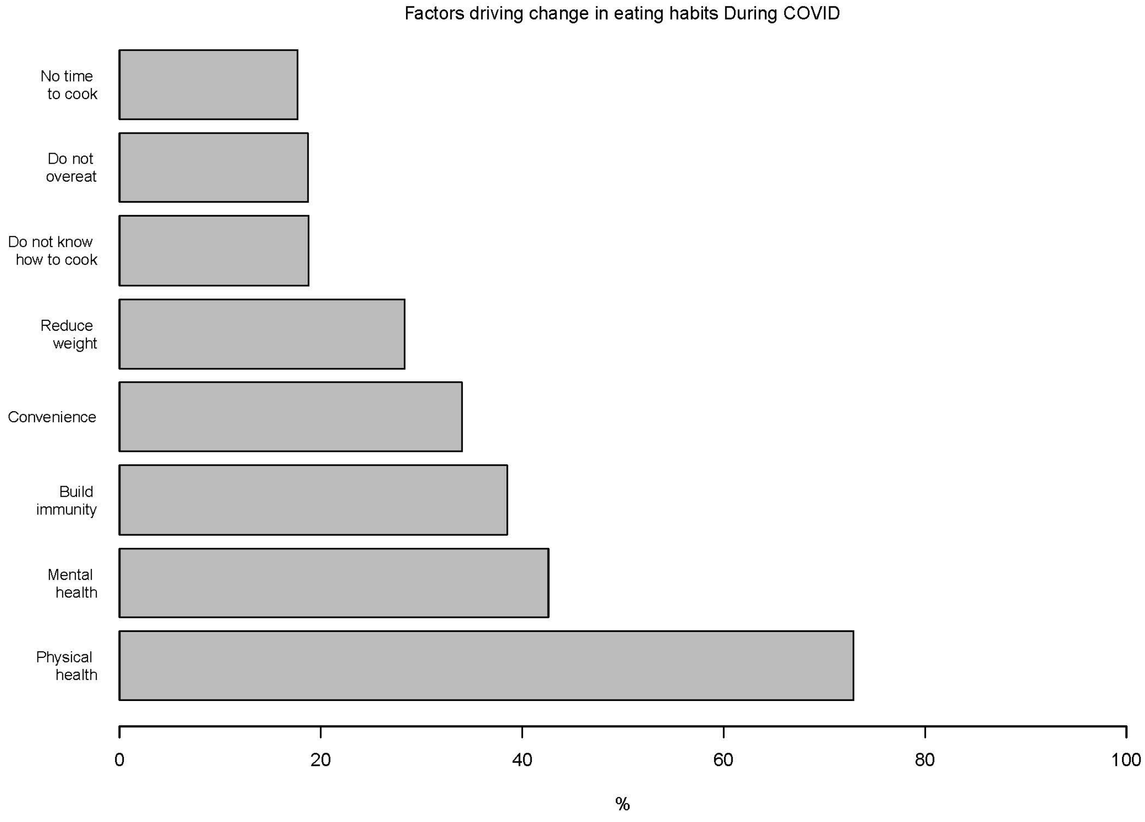

Figure S4. Top factors reported by percentage of participants contributing to a change in dietary habits.

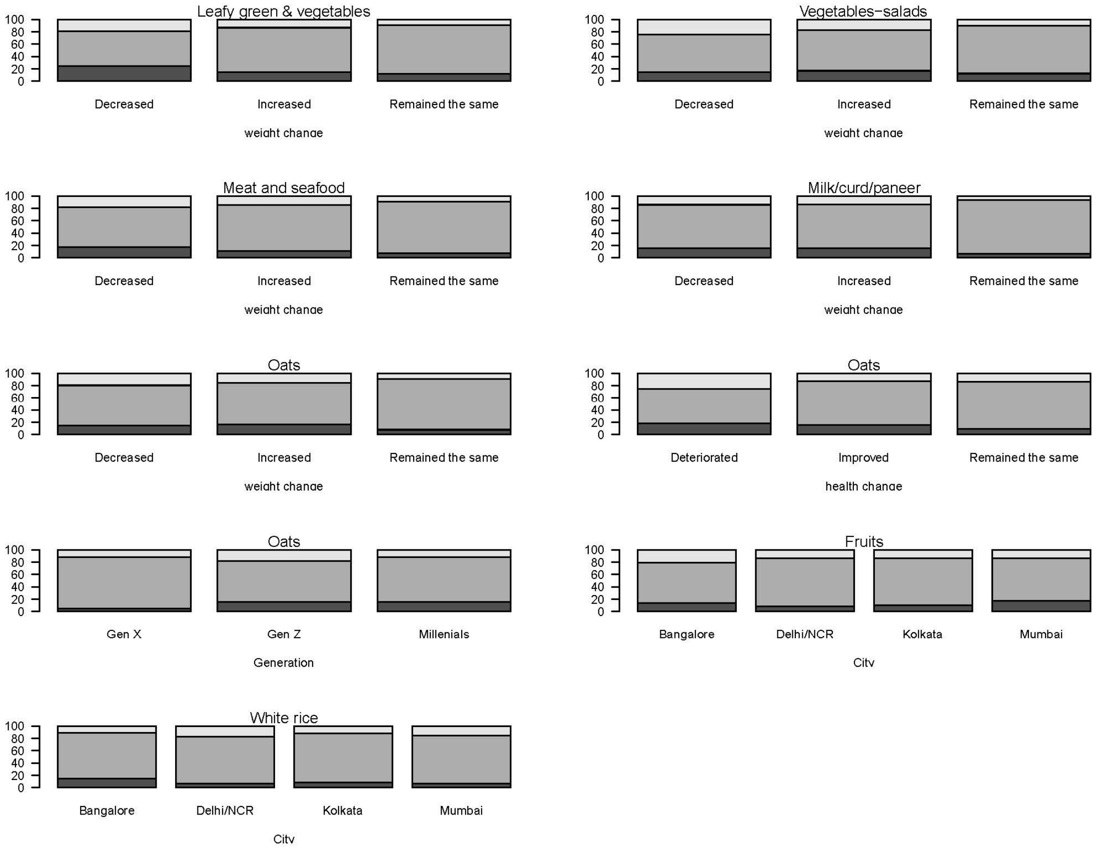

Figure S5. The percentage of subjects reporting a decrease (dark gray), no change (medium-gray), or increase (light gray) during the COVID-19 confinement compared to pre-COVID-19 in dietary intake within self-reported body weight change, perceived health change, generation, and city if significant after multiple comparison adjustments ($q < 0.05$).
